# Electronic data collection to enhance disease surveillance at the slaughterhouse in a smallholder production system

**DOI:** 10.1038/s41598-021-98495-7

**Published:** 2021-09-30

**Authors:** Laura C. Falzon, Joseph G. Ogola, Christian O. Odinga, Leonid Naboyshchikov, Eric M. Fèvre, John Berezowski

**Affiliations:** 1 Institute of Infection, Veterinary, and Ecological Sciences, University of Liverpool, Liverpool, UK; 2 International Livestock Research Institute, Nairobi, Kenya; 3 Veterinary Department, Bungoma County Government, Bungoma, Kenya; 4 Kestrel Technology Group, Sugar Land, TX USA; 5 Veterinary Public Health Institute, University of Bern, Bern, Switzerland

**Keywords:** Infectious diseases, Data acquisition, Preventive medicine, Epidemiology

## Abstract

Globally, meat inspection provides data for animal health surveillance. However, paper-based recording of data is often not reported through to higher authorities in sufficient detail. We trialled the use of an electronic meat inspection form in Kenyan slaughterhouses, in lieu of the currently used paper-based format. Meat inspectors in two ruminant slaughterhouses completed and submitted an electronic report for each animal slaughtered at their facility. The reports, which captured information on the animal demographics and any eventual condemnations, were stored in a central database and available in real-time. A stakeholder meeting was held towards the end of the study. Over the 2.75 year study period, 16,386 reports were submitted; a downward linear trend in the monthly submissions was noted. There was a week effect, whereby more reports were submitted on the market day. Of the slaughtered animals, 23% had at least a partial condemnation. The most frequently condemned organs were the liver, lungs and intestines; the primary reasons for condemnations were parasitic conditions. Lack of feedback and difficulty capturing animal origin information were the primary challenges highlighted. The study demonstrated that electronic data capture is feasible in such challenging environments, thereby improving the timeliness and resolution of the data collected.

## Introduction

Health information generated by surveillance activities can guide disease prioritization, resource allocation, and program implementation strategies^1^. Depending on the scope and setting of the surveillance system, different data sources may be used, including active surveys, diagnostic laboratory reports, medical records, farm observations, and slaughterhouse monitoring programs^2,3^.

While the primary function of meat inspection is to prevent transmission of zoonotic diseases and safeguard food integrity^4^, a number of characteristics make the data collected at the slaughterhouse also suitable for animal surveillance purposes^5^. Slaughterhouses, as centralized structures through which the majority of animals eventually pass in many settings, often have a large catchment area^6,7^. Moreover, it is possible to collect data on diseases which are difficult to detect during clinical examination, such as tuberculosis or fasciolosis, or that require invasive diagnostic tests, such as Bovine Spongiform Encephalopathy^8,9^. Consequently, data collected at the slaughterhouse can be leveraged to provide information on a number of other animal health and welfare issues^7,10–12^. Data from slaughter facilities are, however, underutilised in disease surveillance systems, and may be essential in current moves for integrated One Health surveillance of multi-host pathogens^13^.

**Figure 1:**
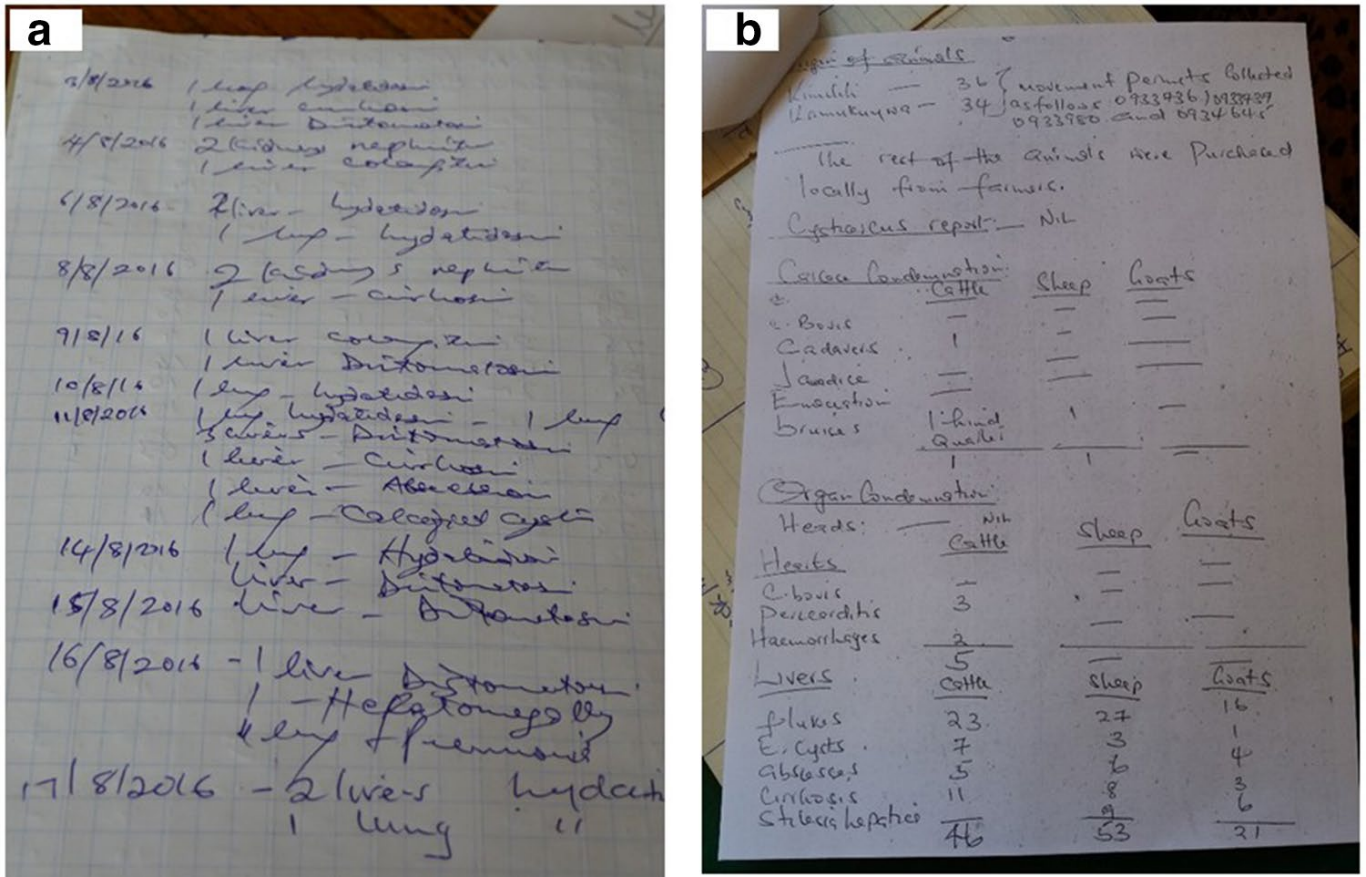
Daily records **(a)** and a monthly compilation **(b)** of condemnations reported in a slaughterhouse in Kenya.

In Kenya, slaughterhouses are categorized as A [large], B [medium] or C [slaughter slabs], based on the animal throughput, infrastructure, and amenities available^14^. Irrespective of the slaughterhouse category, the Meat Inspection Act requires ante- and post-mortem inspection of every slaughtered animal. The post-mortem inspection relies on visualization, palpation, and incision of muscles and viscera to ensure that they are fit for human consumption; carcasses or individual organs that are deemed unfit are to be condemned and disposed of^15^. Meat inspectors are also legally bound to keep daily record books of all animals slaughtered at their facility, together with the number of, and reason for, eventual condemnations. These daily records are then compiled into monthly and annual reports which are submitted to both the County and National Director of Veterinary Services for further evaluation^14^. Currently, both daily records and monthly reports rely on traditional paper-based formats (Fig. 1). This makes reporting labour-intensive and error-sensitive. Furthermore, the aggregation of daily records into monthly reports leads to loss of resolution while also delaying detection of any notable events and, consequently, the response time. Moreover, the data are often collected in an unstructured manner, making it difficult to extract useful information in a timely fashion^12,16,17^.

With the increased affordability and penetration of mobile phones, their potential to contribute to healthcare systems has been increasingly recognized^18^, particularly in low- and middle-income countries where they can improve both the quality and reach of services^19,20^. Digital technologies may assist in various health-related activities, including data collection and surveillance^21–25^. Data can be sent more quickly, reliably, and in a structured manner, compared to paper-based collection methods, thereby facilitating data integration and analysis and allowing for timely and evidence-based decisions^26–28^. In Kenya, the use of mobile technologies to collect near real-time information on syndromes in both livestock and wildlife has been reported^29–31^. However, none so far have explored the use of mobile phones to collect meat inspection data which is statutorily recorded in every licensed slaughterhouse. Obtaining meat inspection data in a timely fashion would help establish baseline information on the number and trend of animals being slaughtered, and the frequency of underlying endemic disease leading to condemnations. Over time, routine slaughterhouse inspection data could also be used to inform thresholds for expected values within an early detection and warning system for emerging diseases and zoonoses of public health concern.

The overall objective of this study, therefore, was to develop and trial an electronic meat inspection form. Specifically in this study we (i) describe the design and implementation of the electronic meat inspection form; (ii) present data on submission patterns during the study period; (iii) provide examples of the data generated on animal throughput and condemnations; (iv) report on stakeholders’ feedback on the form; and (v) describe the costs involved in setting up the system and discuss what it would mean to implement this in practice.

## Results

The initial dataset contained 16,482 reports; however, 58 were removed because they were submitted either before or after the study period. An additional 38 reports were removed either because they were test runs (n = 4), or were for an animal species other than ruminants (n = 2), or did not indicate the animal species (n = 32). If other data entries were missing (e.g. animal age or gender), the report was still retained. Therefore, the final dataset retained for analysis contained 16,386 reports for 7182 cattle, 5495 sheep, and 3709 goats slaughtered at the two facilities between 17th March 2017 and 31st December 2019 (Table 1). This resulted in roughly 16 reports being submitted every day over the study period (8.6 reports and 7.5 reports/day submitted from the Webuye and Kimilili slaughterhouse, respectively).

**Table 1:** The number of ruminants slaughtered, their sex, and the condemnation frequency, overall and stratified by sex, in two slaughterhouses in Bungoma County, western Kenya, between 17th March 2017 and 31st December 2019.

Animal species	No. slaughtered			Sex				Condemnation reported					
	2017	2018	2019	Female	%	Male	%	Total	%	Female	%	Male	%
Cattle	2885	2516	1781	3721	51.9	3449	48.1	2377^a^	33.2	1456	39.1	919	26.6
Sheep	2281	2038	1176	3063	55.8	2428	44.2	1122^a^	20.5	780	25.5	342	14.1
Goat	1902	1142	665	1330	35.9	2370	64.1	278^a^	7.5	141	10.6	137	5.8
Total	7068^a^	5696^a^	3622^a^	8114^a^	49.6	8247^a^	50.4	3777^a^	23.1	2377^a^	29.3	1398^a^	17.0

^a^Total numbers do not add up due to missing data entries for the gender or condemnation variables.

### Number and trend of animals slaughtered

**Figure 2:**
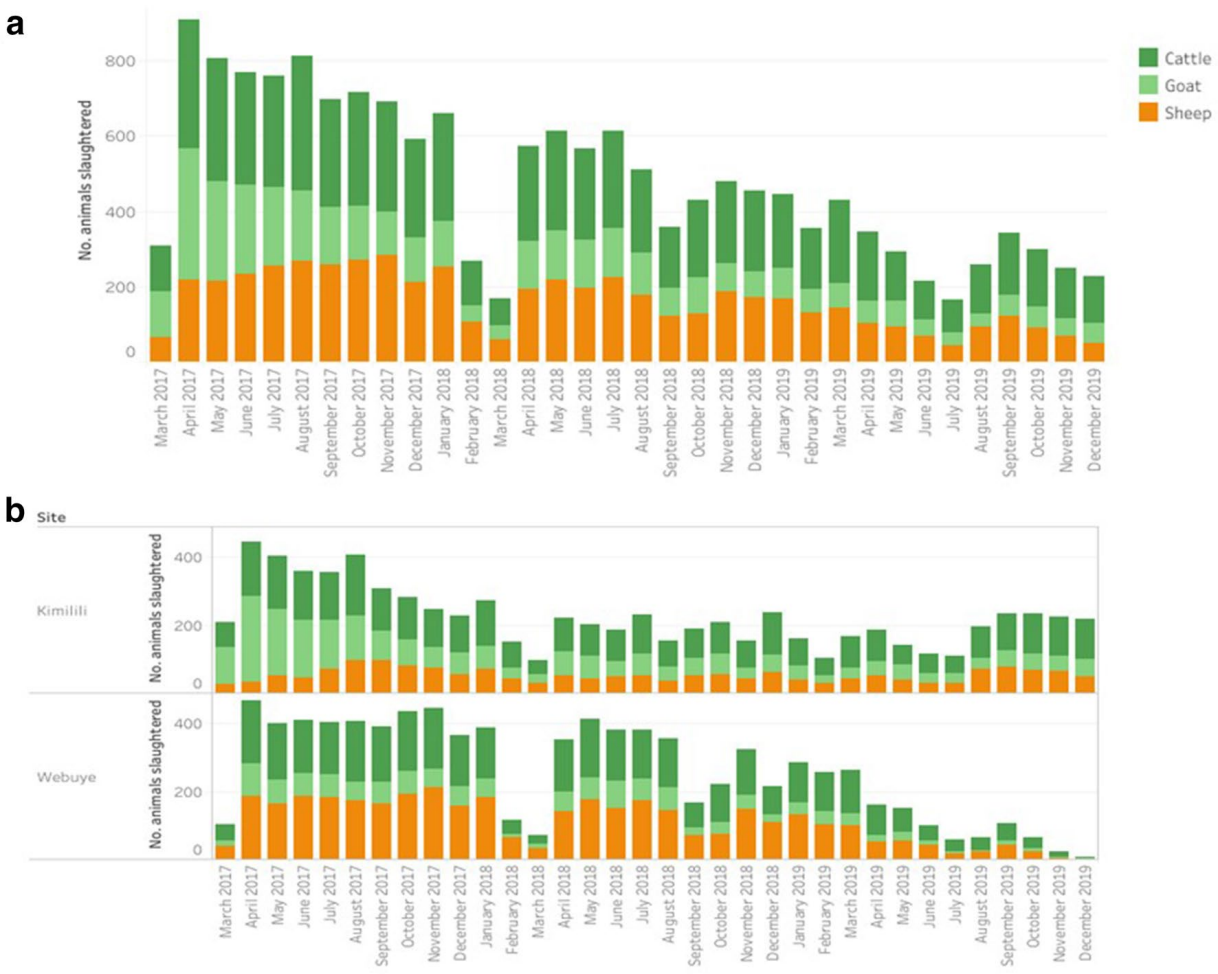
The number of reports submitted by the participating meat inspectors during each month of the study period (March 2017–December 2019) overall **(a)**, and stratified by slaughterhouse **(b)**.

The number of reports submitted decreased over the study period, and this downward linear trend was statistically significant (Spearman’s rank correlation coefficient p-value < 0.001), both overall (Fig. 2a), and for the individual slaughterhouses (Fig. 2b). The decrease in reports submitted, however, was more marked in the Webuye slaughterhouse (trend of −10.62 reports per month), compared to that in the Kimilili slaughterhouse (trend of −5.46 reports per month). No statistically significant seasonal effect was detected.

**Figure 3:**
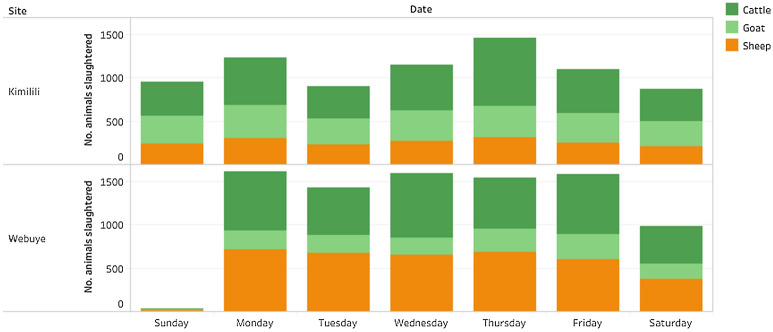
The number of reports submitted on each weekday by the meat inspectors in two slaughterhouses in Bungoma County, western Kenya, between 17th March 2017 and 31st December 2019.

While both slaughterhouses were open seven days a week, there was a statistically significant (p-value < 0.001) week day effect, both overall, and for the individual slaughterhouses (Fig. 3). In Webuye, the number of animals inspected on Sunday was statistically significantly lower than the other days (Beta coefficient for comparisons between Sunday and other week days ranging between −0.93 and −1.03). In Kimilili, the number of animals inspected on Thursdays was statistically significantly higher than the other days (Beta coefficient for comparisons between Thursday and other week days ranging between 0.21 and 0.30).

Almost all the slaughtered animals (n = 16,330; 99.7%) were over 6 months of age. Overall, the number of female and male animals was roughly equal (8114 and 8247, respectively), though more male goats (64.1%) and more female sheep (55.8%) were slaughtered (Table 1). Most of the slaughtered animals were brought from within Bungoma County (n = 16,319), primarily from Webuye (n = 9474), Kimilili (n = 5619), Mount Elgon (n = 792), and Kabuchai (n = 427) sub-counties. The other animals came from Malava sub-county in Kakamega County (n = 37), and Cherangany sub-county in TransNzoia County (n = 1). Thus, these slaughterhouses are mainly sourcing animals from the local area.

### Number and trend of condemnations

Condemnations were reported in 23.1% (n = 3777) of the slaughtered animals (Table 1). This proportion varied depending on the species, with a higher proportion of condemnations in cattle (33.2%), and a lower proportion of condemnations in goats (7.5%). Furthermore, the condemnation frequency was between 1.5 and 1.8 times higher in female animals, compared to male animals, and this difference was statistically significant (Chi-squared p-value < 0.001) in all animal species (Table 1).

Only 38 of these condemnations (1.0%) comprised the full carcass: 25 cattle, 11 sheep, and 2 goats. The most frequent reasons for full carcass condemnation included bruising (n = 11), emaciation (n = 8), and dead on arrival (n = 5). Other reasons for carcass condemnation included: generalized presence of cysts (n = 3), fractures (n = 2), jaundice (n = 2), septicaemia (n = 2), uraemia (n = 1), and dropsy (n = 1); in three instances the reason for the carcass condemnation was not given. The median estimated weight of the dressed cow, goat and sheep full condemned carcasses was 55.0 kg, 9.0 kg, and 8.5 kg, respectively, while the median estimated value was US $130.70, $32.50, and $30.20, respectively.

The majority of the condemnations (n = 3739; 98.99%) comprised one or more organs. The number of organs condemned in each species are presented in Table 2, while the median estimated weight (kg), non-condemned market value (US$), and proportion that were totally (versus partially) condemned, are presented in Table 3. The most frequently condemned organs overall were the liver, lungs, and intestines. A considerable number of foetuses (n = 776) were also discarded during the study period; these have no direct economic value to our knowledge, but do represent potential lost productivity for farmers.

**Table 2:** The number (and overall proportion) of condemnations of each organ, overall and in each animal species, in two slaughterhouses in Bungoma County, western Kenya, between 17th March 2017 and 31st December 2019.

	Total	Cattle	Sheep	Goats
No. slaughtered	16,386	7182	5495	3709
Female animals	8114	3721	3063	1330
**Condemned**				
Liver	2358 (14.4%)	1622 (22.6%)	614 (11.2%)	122 (3.3%)
Lungs	803 (4.9%)	560 (7.8%)	171 (3.1%)	72 (1.9)
Intestines	209 (1.3%)	76 (1.1%)	68 (1.2%)	65 (1.8%)
Kidneys	69 (0.4%)	68 (0.9%)	N/A	1 (< 0.1%)
Spleen	63 (0.4%)	63 (0.9%)	N/A	N/A
Heart	41 (0.3%)	38 (0.5%)	1 (< 0.1%)	2 (0.1%)
Limbs	32 (0.2%)	23 (0.3%)	6 (0.1%)	3 (0.1%)
Head & tongue	11 (0.1%)	10 (0.1%)	1 (< 0.1%)	N/A
Foetus	776 (9.6%)	392 (10.5%)	341 (11.1%)	43 (3.2%)

**Table 3:** The median estimated weight (kg) and non-condemned market value (US$) of each condemned organ, and the proportion that were totally (versus partially) condemned, for each animal species, in two slaughterhouses in Bungoma County, western Kenya, between 17th March 2017 and 31st December 2019.

	Median estimated weight (kg)			Median estimated value (US$)			% Totally condemned		
	Cattle	Sheep	Goat	Cattle	Sheep	Goat	Cattle	Sheep	Goat
Liver	3.0	0.5	0.5	10.9	1.8	1.8	80.3	99.0	95.9
Lung	3.0	0.5	0.2	5.5	0.9	0.6	90.4	99.4	95.8
Intestines	2.0	2.0	2.0	5.5	5.5	5.5	15.6	61.8	73.8
Kidneys	0.5	N/A	0.1	0.9	N/A	0.2	88.2	N/A	100
Spleen	0.5	N/A	N/A	1.8	N/A	N/A	93.5	N/A	N/A
Heart	1.0	0.2	0.1	3.7	0.5	0.1	91.9	100	100
Head	4.0	N/A	N/A	9.1	N/A	N/A	77.8	N/A	N/A

**Figure 4:**
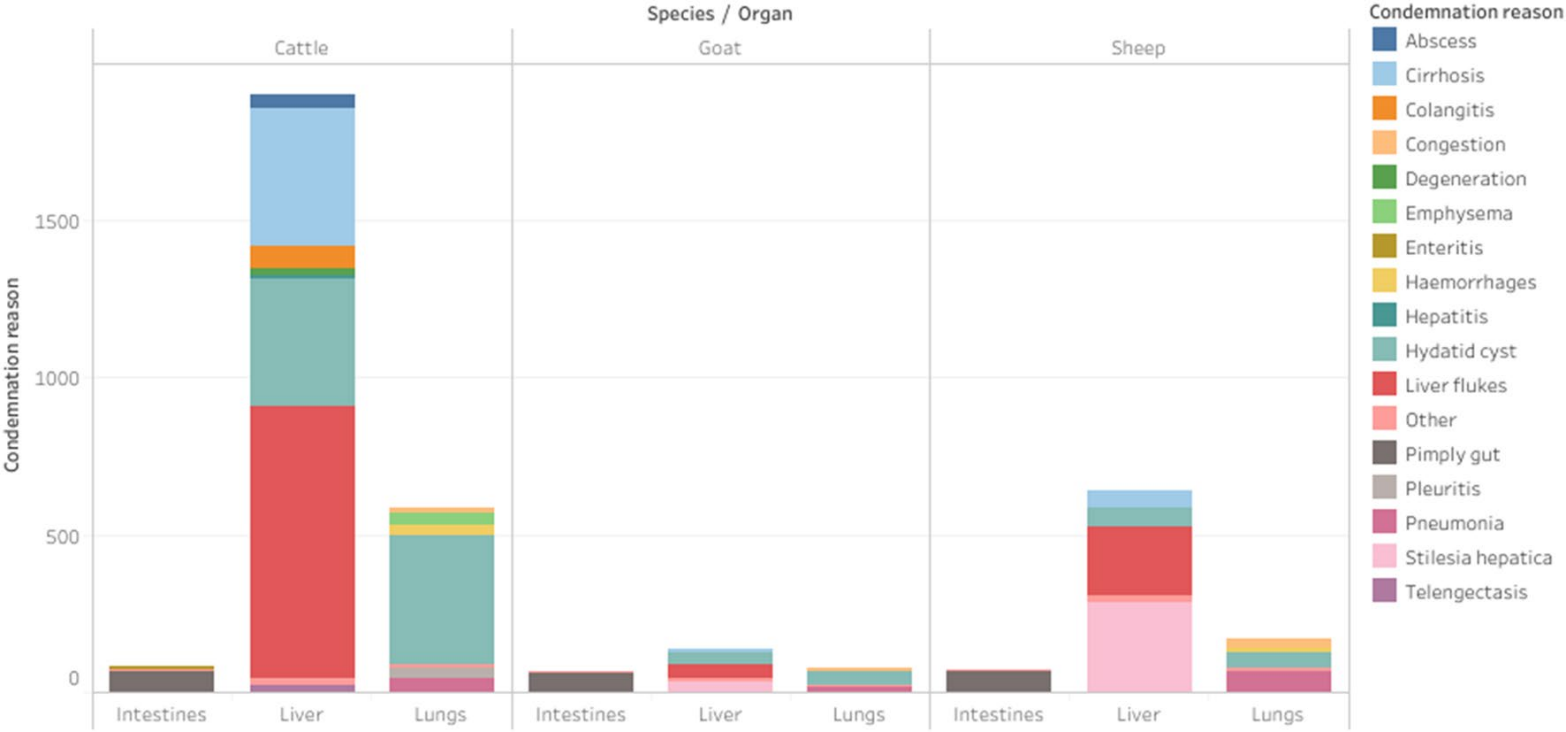
The reasons for condemnations of the intestines, liver, and lungs, and their frequency, in cattle, goats and sheep in two slaughterhouses in Bungoma County, western Kenya, between 17th March 2017 and 31st December 2019.

**Figure 5:**
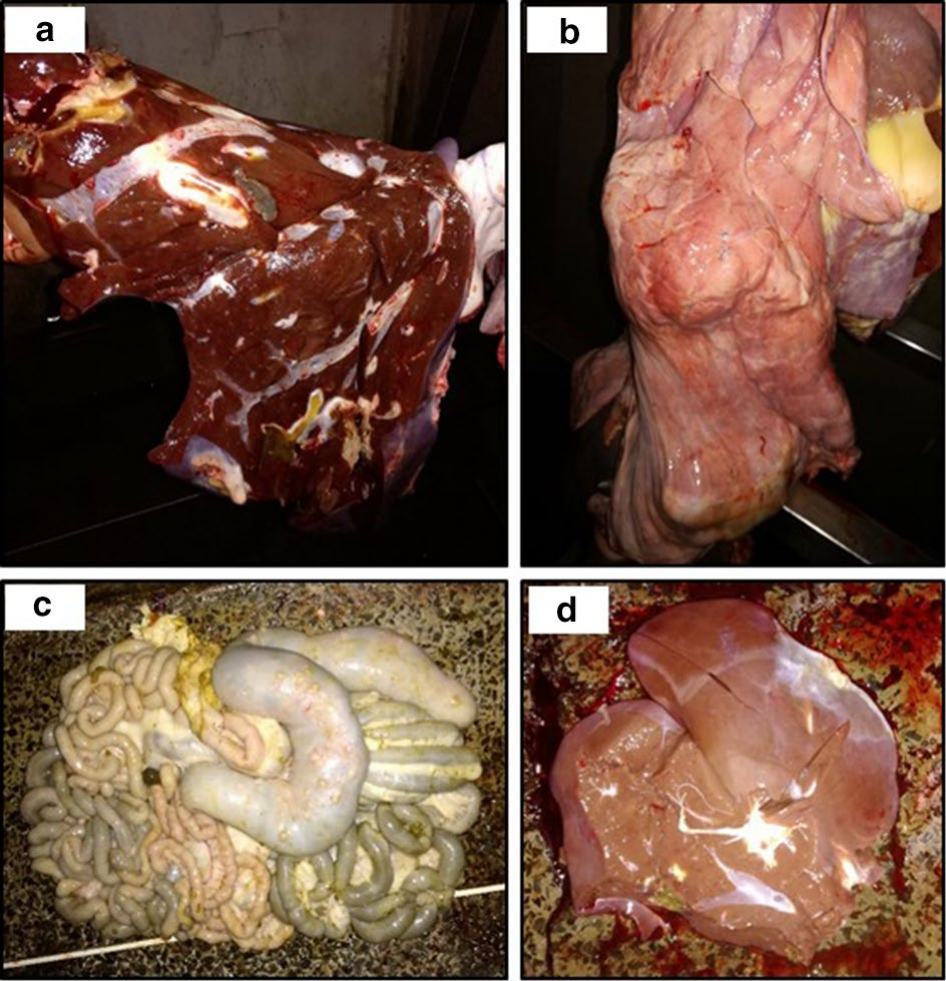
The most commonly reported reasons for condemnation, including liver flukes **(a)**, hydatid cysts **(b)**, pimply gut caused by *Oesophagostomum* spp. **(c)**, and *Stilesia hepatica* **(d)**. These photographs were taken by the meat inspectors and submitted as part of the electronic meat inspection report.

The most frequent reasons for condemnation varied by animal species, with liver flukes being the main cause of liver condemnations in cattle (866/1622; 53.4%) and goats (47/122; 38.5%), followed by either cirrhosis (436/1622; 26.9%) or hydatid cysts (39/122; 32.0%) in cattle and goats, respectively. On the other hand, the leading cause of liver condemnations in sheep was *Stilesia hepatica* (283/614; 46.1%), followed by liver flukes (218/614; 35.5%). Similarly, hydatid cysts (caused by *Echinococcus granulosus* spp.) were the leading cause of lung condemnations in both cattle (414/560; 73.9%) and goats (42/72; 58.3%), while pneumonia was the leading cause of lung condemnations in sheep (67/171; 39.2%). On the other hand, pimply gut (caused by *Oesophagostomum* spp.) was the main reason for condemnation of the intestines in all three animal species (cattle: 66/76; 86.8%; goats: 59/65; 90.8%; sheep: 66/68; 97.1%) (Figs. 4 and 5).

Reasons for condemnation of the other organs varied in all three species. Overall, the kidneys were condemned primarily because of nephritis (n = 35), hydronephrosis (n = 12), hydatid (n = 9) or congenital cysts (n = 7). The spleen was also primarily condemned because of hydatid cysts (n = 35), followed by splenomegaly (n = 10), abscesses (n = 9), and congestion (n = 5). Pericarditis was the leading cause of heart condemnations (n = 27), followed by *Cysticercus bovis* (n = 8), haemorrhages (n = 2), myocarditis (n = 2) and hydatid cysts (n = 2). Finally, the head and tongue were condemned either because of abscesses (n = 6) or because of the presence of *C. bovis* cysts (n = 3).

In a number of animals, multiple organs were condemned. Both livers and lungs were condemned in 374 animals, and in 51% of these animals (n = 190) the reason was due to hydatid cysts present in both organs. Other frequent combinations included condemnations of the liver and the intestines (n = 46), and condemnations of the liver, lungs and spleen (n = 34). In the latter case, hydatid cysts were responsible for 88% (n = 30) of these condemnations.

**Figure 6:**
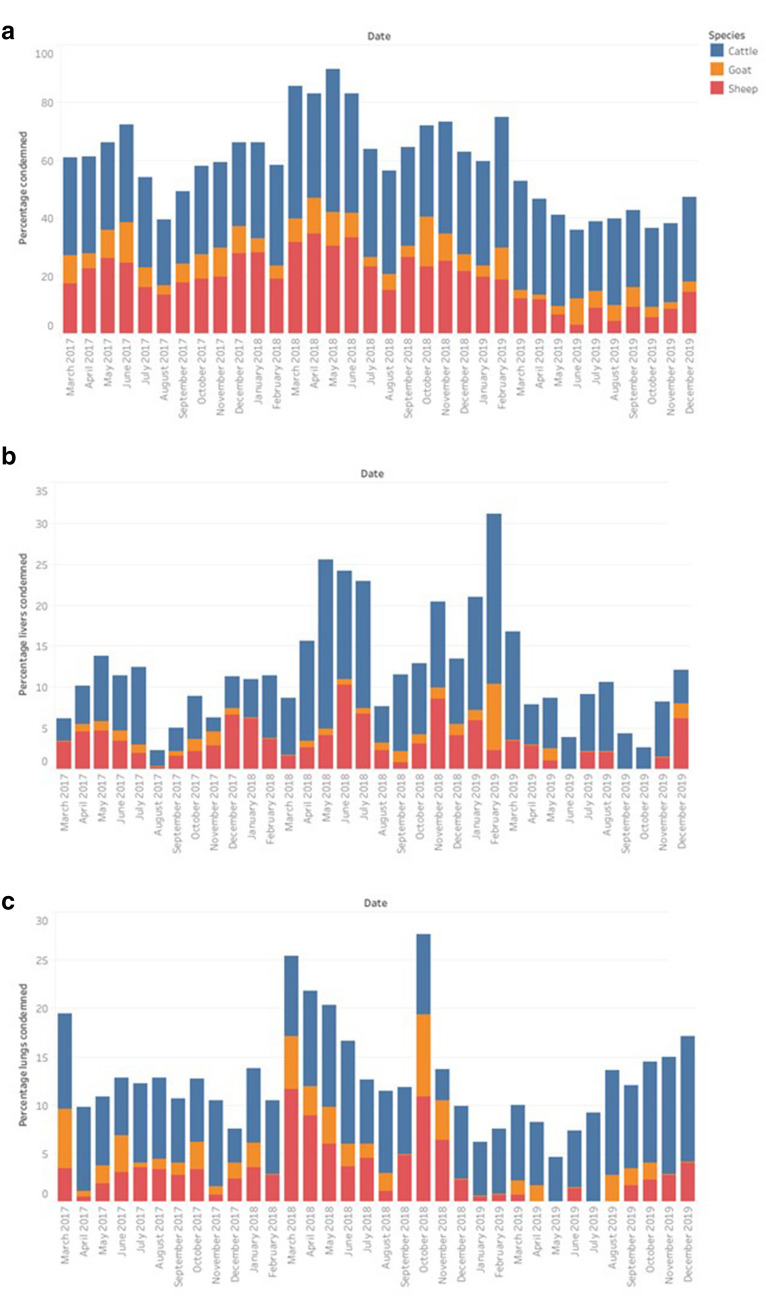
The percentage of condemnations **(a)**, and the percentage of livers **(b)** and lungs **(c)** condemned in two slaughterhouses in Bungoma County, western Kenya, between 17th March 2017 and 31st December 2019.

The monthly trend of the percentage of all condemnations in each slaughterhouse, and of the liver and lung condemnations, is illustrated in Fig. 6. Both the liver and lung condemnations showed a statistically significant downward trend (Spearman’s rank correlation coefficient p-value < 0.001), with −1.84 and −0.65 condemned livers and lungs, respectively, each month. However, there was no trend in the percentage of all condemnations, or the percentage of liver and lung condemnations, per month. No seasonal effect in the number of liver and lungs condemned was detected.

### Electronic form evaluation

During a stakeholder feedback meeting held in April 2019, preliminary study findings were presented to the stakeholders. A discussion on the advantages and the challenges encountered when using the electronic data collection form ensued.

One of the participating meat inspectors found the process time-consuming, estimating that it took roughly 30 min every day to complete and submit the forms. However, when we suggested that one of the options to make the process shorter was to only take photos of condemnations (and not for every slaughtered animal), all stakeholders disagreed with this. The meat inspectors felt that they had now integrated the photo-taking within the process and were not keen to change, while the vet officers believed that the photos served as an indicator of veracity.

It was suggested that the form could be modified to capture data on conditions that do not lead to condemnation, such as pulmonary emphysema and atelectasis, and on conditions of inedible portions of the animal, such as the hide and skin. Another suggestion was to modify the form to also include data on the ante-mortem inspection. However, it was recognized that while the ante-mortem inspection is required by law, it is often difficult to carry out due to staff shortages and pressure from butchers and meat traders.

“[…] at times it is very hard for meat inspectors to do the ante-mortem inspection. That is now from the practical point of view. It is in the training and the legislation says you need to do ante-mortem, but you know that only works well if you are two. Assuming you are one and this meat is supposed to move to another place. Assuming you are one, so if I could be there in time and inspect these animals as they come and they go in now, I need to follow them. That is now the challenge in the meat inspection”.

A discussion ensued on how the situation could be resolved. The county and sub-county vet officers felt that the meat inspectors should be more rigorous and enforce the legislation. The meat inspectors, however, argued that they needed more support from their supervisors to face up to the butchers and meat traders. They also suggested introducing heftier fines for those who are caught selling uninspected meat.

Another salient issue was the origin of the animal. While the electronic form allowed for the collection of this information, this referred to the animal’s last destination and did not capture previous movements. This was particularly relevant for certain conditions such as hydatid cysts due to cystic echinococcosis, which is traditionally known to be present in the Turkana and Pokot counties in the northern part of Kenya. The participants thought it likely that animals with hydatid cysts are coming from these areas, but it is currently hard to map the origin and progression of this disease as only their last known location (e.g. market) is recorded on movement permits.

“Most of the animals with hydatid cyst come from far. But for you to determine where they come from it’s very tricky […]”.

One of the meat inspectors suggested liaising with auction officers at the livestock markets to glean information on the animal’s movement history.

The meat inspector who had not participated in the study and who worked in a Category C slaughter slab highlighted how he felt that it would be difficult to implement the system at his facility which is less structured:

“[…] my slaughter slab is more or less like a hall than a slaughterhouse so I don’t even have space to take that [phone with electronic form]. Where they slaughter from is where everything is done, so it is difficult […]”.

One of the participating meat inspectors highlighted the importance of receiving feedback, not only in terms of summaries of the data they themselves submitted, but also as an opportunity to learn.

“[…] it should be sort of an exchange of ideas so that when I post you something and indicate I am not also clear with it, maybe with this modern technology I expected feedback immediately so it also sharpens me [...]”.

The meat inspector also suggested the possibility of connecting with a laboratory so they can get confirmation of their presumptive findings.

Overall, the consensus was that the system provided them with an empowering opportunity to learn. They encouraged us to share our study findings with the Directorate of Veterinary Services and Public Health Authorities, as they felt it would be good if the government adopted the system to help inform evidence-based decisions.

### Costing of the system

Setting up the system described in this study involved a one-off cost of purchasing the mobile phone for each meat inspector (£220; US$303), and a standing cost of purchasing monthly bundles for the meat inspectors (1000 KES; US$9.26). Additionally, there was a one-off cost in developing the electronic form which can now be modified based on the feedback received and used by others. All these costs were covered by project grants, as described in the funding section.

In Kenya, there currently are 2100 registered slaughterhouses (120 Category A, 280 Category B, and 1700 Category C). Therefore, based on the costs incurred in this study, rolling out this system nation-wide would require a one-off cost of US$636,300 to purchase mobile phone for all meat inspectors, and a standing cost of US$19,446/month to purchase data bundles. Furthermore, there might be additional costs involved in setting up a centralized system where data are received, collated, analysed, and disseminated. This, however, would also be a one-off cost which could be incorporated within existing national surveillance systems.

## Discussion

This study illustrated how a mobile phone-based meat inspection form could facilitate data reporting and improve extraction of animal health information. More generally, these data could be integrated within the national surveillance system to provide updated estimates of the frequency of endemic diseases; a broader rollout of digital tools for animal health surveillance is underway in Kenya^32^. This information could then help authorities identify which campaigns to run to improve animal health and welfare. Furthermore, as baseline data are established over time, the observed spatial and temporal trends could be quantified and projected forwards to help improve preparedness for future disease outbreaks. These data could also lay the groundwork for a syndromic surveillance system, whereby a higher than expected condemnation rate could raise the alarm about possible disease events or welfare concerns in the area.

A total of 16,386 reports were submitted by the participating meat inspectors over the study period, roughly 8 reports per day per slaughterhouse. This corresponds to the expected throughput of Category B [medium] slaughterhouses, which ranges between 6 and 39 cattle, or 16 and 24 small ruminants^14^. However, a downward linear trend in the number of submitted reports was noted over the duration of the study period (Fig. 1). Both slaughterhouses had to close for a while in February–March 2018 because of water shortages [Webuye] and a Foot-and-Mouth Disease outbreak [Kimilili]. During the feedback meeting, some of the stakeholders also indicated that 2018 was a bad crop season, particularly for maize and beans which are an important source of income in Bungoma County. This led to cash shortages and a diminished demand for meat, resulting in fewer slaughtered animals. In the Webuye slaughterhouse a notable decrease in reports was noted after May 2019 (Fig. 1b). As mentioned in the Methods section, the research team was visiting each participating slaughterhouse every month as part of another research project in the same study area^13^. However, this study was completed in June 2019, and the research team was less available to follow-up on any difficulties the meat inspectors might have encountered after that date. This highlights the importance of providing ongoing technical support and mentorship of participants to ensure that they remain confident using the tools^21,26^. This is particularly important for older inspectors who may be less digitally literate, and who may also be more reluctant to adopt new methods, though training and acquisition of familiarity with the tool can help overcome these challenges^16,17,22^. Ensuring that data are regularly fed back to data collectors would also be key in ensuring continued compliance. Furthermore, completing the electronic forms in this trial did not remove the statutory requirement to fill the paper forms, so we were effectively increasing the workload of the participating staff. A national adoption of e-surveillance tools would dispense with this requirement.

A week of the day effect was observed in both slaughterhouses (Fig. 3). Sundays were less busy days, particularly in the Webuye slaughterhouse, while in Kimilili more animals were slaughtered on Thursdays. This was not unexpected since Thursdays are the market days in Kimilili, when butchers from other areas will come to the livestock market to buy animals and have them slaughtered in the nearby facility, to then transport the meat. Hence more animals are usually brought to slaughter on market days. These socio-economic factors should therefore be taken into consideration when setting expected trends and thresholds within predictive disease models or syndromic surveillance systems. On the other hand, we were unable to detect a statistically significant seasonal effect in the number of reports submitted over time, though this could be due to the relatively short duration of the study period.

Almost a quarter of all slaughtered animals had some form of condemnation (Table 1), though this varied by species, with a higher condemnation rate (33.2%) in cattle, and a lower condemnation rate (7.5%) in goats. These differences in condemnation rates between the species could be attributable to different eating habits since goats, unlike cattle and sheep which graze, are browsers and are therefore less likely to be exposed to pathogens such as liver flukes^33^. The condemnation frequency was also higher in female (29.3%), compared to male (17.0%), animals. This statistically significant difference might be due to age, since female animals are usually kept for breeding and slaughtered at an older age, compared to male animals. However, in this study we were unable to explore this further since age was categorized as either younger than 6 months, or 6 months or older, with almost all animals classified in the latter category, thus limiting our capacity to explore the effect of age on condemnations.

Out of the 3777 condemnations reported by the participating meat inspectors, only 38 (1.0%) involved the full carcass; the majority of the condemnations (99.0%) therefore comprised total or partial condemnation of one or more organs. The most frequently condemned organs were the liver, followed by the lungs and the intestines (Table 2). The most common causes of condemnation—fasciolosis, echinococcosis, pimply gut caused by *Oesophagostomum* spp., and *Stilesia hepatica* (Fig. 5)—are similar to those reported by studies carried out in other Kenyan slaughterhouses^34,35^. These conditions might be over-represented in a slaughterhouse study population due to volunteer bias, whereby farmers are more likely to sell those animals that they suspect to have a disease, to avoid the losses that would occur if they waited until the animals became emaciated. During the feedback meeting, the meat inspectors also noted how the leading causes of condemnation were all parasitic in nature. They recalled how in the recent past improved sanitation had helped to control another parasitic disease, *Cysticercus bovis*, demonstrating that it is feasible to control these other diseases of economic importance through targeted interventions and public health education.

Liver flukes were responsible for 48.0% of all the reported liver condemnations, and for 30.2% of all organ condemnations, highlighting the high prevalence and consequent economic burden of fasciolosis in the study area. These findings are in agreement with a study by Kithuka et al.^36^, which found that the former Western Province [including Bungoma County] had the highest mean prevalence of fasciolosis among all Kenyan provinces. This high prevalence could be attributable to the high rainfall experienced in the area, and the large population of extensively managed livestock in the area, thus perpetuating the disease^36^. Indeed, increased rainfall, together with higher temperatures, have been associated with increased liver condemnation due to fasciolosis^8^, and the environment in western Kenya supports significant populations of snail intermediate hosts infected with *Fasciola* spp.^37^. Therefore, the integration of climatic data within future animal health surveillance systems could aid control of fasciolosis.

The second most frequent cause of condemnations were hydatid cysts, which were responsible for 21.6% and 63.0% of all the reported liver and lung condemnations, respectively. Furthermore, hydatid cysts often led to the condemnation of multiple organs, as also reported by Froyd et al.^38^, further exacerbating the economic impact of this disease. Hydatidosis, or cystic echinococcosis, is a neglected but re-emerging zoonotic disease caused by larval stages of the tapeworm *Echinococcus granulosus *sensu lato^33^. The disease is known to be endemic in Kenya, with high prevalence reported in transhumant pastoralist communities in Turkana (northwest) and Maasai (south)^39,40^. However, cattle rustling and the constant migration of animals from these high-risk areas to areas with high demand for meat products may lead to the disease becoming established in other areas across the country^36,40^. As mentioned by the meat inspectors during the feedback meeting, obtaining information on the origin of the animal would shed light on whether this disease is being continuously imported from other high-risk regions, or whether it has also become endemic to the region. Identifying the original animal owners would also facilitate feedback of information from the meat inspection, thus alerting them about potential parasite and other disease problems in their herd. This, however, can be challenging since animals are usually brought to the slaughterhouse by intermediate traders who may therefore not be familiar with the animal’s full movement history^3,41^. Consequently, liaising with other stakeholders, such as auction officers, and integrating different data sources including animal identification and movement history, is essential for improving animal traceability and disease tracking^42^.

The electronic form used in this study also captured information on the weight and non-condemned market value of any organ condemned, together with whether it was totally or partially discarded (Table 3). These data are not reported in the paper-based forms but were included in the electronic form following a request by the participating meat inspectors. Generating information on the monetary impact of the leading causes of condemnation and underlying diseases can improve estimates of their economic and social impact, thus aiding priority setting and implementation of pathogen-reduction measures^5,43^. Furthermore, regular monitoring of the prevalence and production losses of these diseases can also contribute towards estimating their overall global burden^44^.

Almost 10% of all the slaughtered female animals were pregnant (Table 2), and this may be an under-estimation since early pregnancies are harder to detect and may therefore go unnoticed. Of the 776 pregnant animals, 19% (n = 147) had concurrent condemnations; the remaining 81% had no condemnations, further highlighting the significant lost production of these animals. While some pregnant animals are knowingly sent to slaughter due to health concerns or out of economic necessity, it is likely that in this context the owners were often unaware that the animal was pregnant. This occurs due to poor record-keeping on the farm or loss of information when animals are traded at markets. Besides the animal welfare concerns this may raise if the slaughtered animals are in advanced gestational stages, slaughtering pregnant animals is also an uneconomical practice as it leads to both productive and reproductive losses. Loss of a calf in such traditional smallholder production systems, which are characterized by high mortality and low fertility rates, is important. In these herds, the average mean age at first calving is 47.9 months, while the overall mortality risk for calves (0–12 months) and female replacement stock (> 12 months to parturition) is 21.7% and 8.5%, respectively; the mean calving rate is 58.7%^45^. This results in there being virtually no spare female calves, with little or no cattle population growth; this situation is then further aggravated when drought hits. Therefore, when farmers unknowingly rid themselves of animals which are pregnant, they are losing not only productivity in terms of meat and other animal products, but also a potential source of capital^46^. Providing training for farmers and animal health workers on farm record-keeping and simple methods of pregnancy diagnosis could reduce the needless slaughter of pregnant animals^47–51^.

During the feedback meeting, one of the meat inspectors commented that it took roughly 30 min to submit the daily reports. We recognize that this is rather onerous, particularly since, as previously mentioned, they currently also need to keep the paper-based daily activity book mandated by the Meat Inspection Act^14^. However, if the electronic form piloted in this study were to replace the paper-based forms, we are confident that the reporting process would become less time-consuming. One of the major limitations of our study was the lack of feedback provided, as also highlighted by one of the participating meat inspectors. During the inception of the study, we planned to provide monthly summary reports of the data they had submitted. However, due to logistical and technical challenges, we were unable to do so during the study period. We recognize, though, that lack of regular feedback can lead to the participants’ loss of interest and consequent under-reporting^52,53^. Furthermore, a major advantage of digital technologies is that they allow for bi-directional information sharing, thus improving participation by motivating and empowering those who are submitting the data^22^. This facility should therefore be capitalized on to improve engagement of all participating stakeholders, and it is our priority that such a feedback system is incorporated in any future roll-out of this form.

While the slaughterhouses participating in this study were selected purposively, we believe they are broadly representative of all Category B slaughterhouses in the country with respect to acceptability and potential value of electronic data recording. The electronic form could therefore easily be incorporated within the daily routines of meat inspectors working in such slaughterhouses. Based on our approximate estimation, rolling out the system nation-wide would require a one-off cost of US$636,300 to purchase mobile phones for all meat inspectors, and a standing monthly cost of US$19,446 to purchase data bundles. These costs, however, might be over-estimated since fewer data bundles compared to the ones used in our study might suffice. Furthermore, some of the smaller slaughterhouses are manned by the same meat inspector, and there is currently an ongoing discussion to centralize slaughterhouses, which would reduce the number of operating slaughterhouses. Recently, a similar antimicrobial resistance surveillance strategy has been implemented in Kenya, indicating that implementing such surveillance systems is possible in practice through the involvement of all relevant stakeholders, including the meat inspectors and their training institute, the County Veterinary departments, and the national veterinary authorities.

The electronic form used in this study was successful in demonstrating to the meat inspectors and veterinary authorities that electronic data capture is feasible in such challenging environments. Moreover, the form allowed for instantaneous reporting on every animal slaughtered, thus improving the resolution of the data compared to the currently used paper-based forms. By enabling real-time data collection and sharing, the use of this form could also minimise errors and under-reporting, thus improving both the quality and efficiency of reporting. Meat inspection data could therefore become a valuable data source for surveillance of endemic and epidemic diseases which continue to pose a large burden on poor communities, despite the availability of cost-effective control strategies. As baseline data are established over time, meat inspection reporting could also contribute to a syndromic surveillance system, allowing for early detection of any anomalies in condemnation frequency. Furthermore, the meat inspection data could be amalgamated with other surveillance components or data sources to enhance the coverage and effectiveness of the surveillance system and contribute towards the establishment of a national syndromic surveillance system in livestock. Our findings in this regard are relevant to Kenya but also all other livestock-driven economies in the region.

## Methods

### Study site

Two ruminant slaughterhouses in Bungoma County, western Kenya, were included in this study: one was located in Kimilili sub-County and had one meat inspector, while the second slaughterhouse was located in Webuye sub-County and had two meat inspectors. Both slaughterhouses are classified as Category B [medium] slaughterhouses based on their animal throughput and infrastructure^14^, and operate daily from 7am until noon. The two slaughterhouses were selected purposively based on the good working relationship established with the meat inspectors who were also participating in an integrated surveillance programme for zoonotic diseases^13^.

### Form development and data submission

Meetings were held with the participating meat inspectors to discuss which data they routinely collect or would like to investigate further. This information was then used to develop an electronic meat inspection form using the Field Information Support Tool (https://ktg-tech.com/). The form consisted primarily of single-answer multiple-choice questions, but also allowed for photographs and additional comments in free-text. Data collected included: the meat inspector submitting the form; the species, age, sex, physiological status and origin of the animal; and, when applicable, the organ(s) condemned, the reason(s) for the condemnation, whether it was a total or partial condemnation, and the estimated weight and market value of the condemned part(s). The geo-location, date, and time of each submitted report were also captured.

In this study, the definition and consequent actions of condemnation were based on the Kenya Meat Inspection Act^14,15^, which is the guiding document for all slaughterhouse activities. Condemnation refers to the permanent disposal of a carcass, organ, or part thereof, which is deemed unfit for human consumption. The condemnation options available to the meat inspector include: (i) total carcass condemnation (e.g. in the case of notifiable diseases, such as anthrax, or in the case of generalized or acute septic conditions); (ii) total organ condemnation (e.g. abscesses or inflammation of an organ which alters the structure and appearance of the organ); and (iii) partial organ condemnation (when the lesions are slight and localized, the structure and appearance of the organ is not altered, and adequate trimming can be carried out)^15^.

The form was uploaded onto GPS-enabled Android mobile devices which were given to the meat inspectors during a one-day training course where they were trained to complete and submit the form. The meat inspectors were then asked to submit a few test reports to identify any errors or discrepancies in the form, and revisions were carried out as needed. From the 17th of March 2017 until the 31st of December 2019, the meat inspectors were asked to complete and submit a report for each animal slaughtered at their facility; during this period the meat inspectors were still obliged to keep the paper-based daily activity book mandated by the Meat Inspection Act^14^. The electronic data reports were transferred automatically and directly into an electronic custom designed database managed by Kestrel Technology Group, LLC. The meat inspectors were provided with monthly data bundles of 1000 KES (US$9.26) throughout the study period. Up until June 2019 we were visiting each slaughterhouse once a month as part of another project^13^, and during this time we could follow up with the meat inspectors and troubleshoot any problems they encountered. During these monthly visits we also gave feedback on findings from the ongoing surveillance study^54^. We were also available by phone throughout the entire study period.

### Data cleaning and analysis

Data were downloaded from the electronic database as a .csv file and data cleaning was carried out. MobaXterm software was first used to identify and correct minor errors in data entries, and to split data columns with multiple responses. Microsoft Excel (Microsoft, Redmond, WA, USA) was then used to identify missing or inconsistent data entries.

Descriptive statistics to summarize continuous and categorical variables as median values and percentages, respectively, were carried out using Stata Statistical Software:Release 14 (College Station, TX: StataCorp LP). Time-series were first developed in Tableau Online Server Version:2020.4.0 to visualize trends in the weekly and monthly counts of animals slaughtered and organs condemned. Next, the monthly trend, seasonality, and weekday effect, were modelled in R version 4.0.3^55^. Monthly trend and seasonality were estimated using the *decompose()*, *tlsm()* and *Acf()* functions in the *forecast* package^56^. Statistical significance of linear trends in monthly data were estimated using the *cor.test()* function. To model the differences between weekdays, Poisson generalized linear models were fit to data that were not over dispersed [Kimilili slaughterhouse] using the *glm()* function. Over dispersion was considered to be present when the model residual deviance divided by the model degrees of freedom exceeded 1, and confirmed with the *dispersiontest()* function from the *AER* package (https://CRAN.R-project.org/package=AER)^57^. When over dispersion was present [Webuye slaughterhouse], a negative binomial model was fit to the data using the package *MASS* (https://www.stats.ox.ac.uk/pub/MASS4/)^58^. To assess model fit, the final models were compared to the null model using a likelihood ratio test *lrtst()* from the *lmtest* package^59^. Weekday counts were compared using a Tukey method for multiple contrasts in the package *multcomp*^60^.

### Study evaluation

In April 2019, a feedback meeting with eight stakeholders, including four meat inspectors (the three who participated in the study and another one from a neighbouring Category C slaughter slab), three sub-county veterinary officers, and one county veterinary officer, was held. Preliminary study findings were presented to all participants, and a semi-structured discussion on their experiences using the data collection form and submission process ensued. This discussion was recorded, transcribed, and key findings summarized.

### Ethical approval

This study was approved by the Institutional Animal Care and Use Committee (IACUC Reference No. 2017-04) and the Institutional Research Ethics Committee (IREC Reference Nos. 2017-08 and 2015-10/3) at the International Livestock Research Institute, review bodies approved by the Kenyan National Commission for Science, Technology and Innovation, and also approved by the Federal wide Assurance for the Protection of Human Subjects in the USA, and all methods were performed in accordance with the relevant guidelines and regulations. Approval to conduct the work was also obtained from the Kenyan Department of Veterinary Services, and the relevant offices of these Ministries at devolved government level, and all those who participated in the feedback meeting gave written, informed consent prior to their participation.

## Data Availability

All data have been uploaded to the University of Liverpool repository and are now available at the following link: https://doi.org/10.17638/datacat.liverpool.ac.uk/1400.

## References

[1] Dowell SF, Blazes D, Desmond-Hellmann S (2016). Four steps to precision public health. Nature.

[2] Institute of Medicine and National Research Council. *Sustaining Global Surveillance and Response to Emerging Zoonotic Diseases* (ed. Keusch, G.T. *et al*.) 1–312 (The National Academies Press, 2009). 25009943

[3] Hattendorf, J., Bardosh, K.L. & Zinsstag, J. One Health and its practical implications for surveillance of endemic zoonotic diseases in resource limited settings. *Acta Trop*. **165,** 268–273 (2017). 10.1016/j.actatropica.2016.10.009 27769875

[4] Bell RG (1993). Development of the principles and practices of meat hygiene: A microbiologist’s perspective. Food Control.

[5] Lynch JA, Silva P (2013). Integrating animal health and food safety surveillance data from slaughterhouse control. Rev. Sci. Tech..

[6] Schärrer S (2015). Cost and sensitivity of on-farm versus slaughterhouse surveys for prevalence estimation and substantiating freedom from disease. Prevent. Vet. Med..

[7] Correia-Gomes C (2016). Pig abattoir inspection data: Can it be used for surveillance purposes?. PLoS ONE.

[8] Innocent GT (2017). Combining slaughterhouse surveillance data with cattle tracing scheme and environmental data to quantify environmental risk factors for liver fluke in cattle. Front. Vet. Sci..

[9] Mazeri S, Rydevik G, Handel I, de Bronsvoort BMC, Sargison N (2017). Estimation of the impact of *Fasciola hepatica* infection on time taken for UK beef cattle to reach slaughter weight. Sci. Rep..

[10] Stärk KDC (2017). Abattoir condemnation data remain under-used in decision-making. Vet. Rec..

[11] Stärk KDC (2014). Strengths and weaknesses of meat inspection as a contribution to animal health and welfare surveillance. Food Control.

[12] Vial F, Reist M (2014). Evaluation of Swiss slaughterhouse data for integration in a syndromic surveillance system. BMC Vet. Res..

[13] Falzon LC (2019). One Health in action: Operational aspects of an integrated surveillance system for zoonoses in western Kenya. Front. Vet. Sci..

[14] CAP. 356. *Meat Control (Local Slaughterhouse) Regulations, 2010 [Rev. 2012]* 53–70 (2010).

[15] CAP. 356. *Meat Control (Slaughterhouse) Regulations, 1973 [Rev. 2012]* 9–26 (1973).

[16] Madder M (2012). e-Surveillance in animal health: Use and evaluation of mobile tools. Parasitology.

[17] Hutchison J (2019). New approaches to aquatic and terrestrial animal surveillance: The potential for people and technology to transform epidemiology. Prevent. Vet. Med..

[18] Ravindran S (2021). Smartphone Apps test and track infectious diseases. Nature.

[19] National Academies of Sciences, Engineering, and Medicine. In *Using Technology to Advance Global Health: Proceedings of a Workshop* (ed. Taylor, R.M. & Alper, J.) 1–97 (The National Academies Press, 2018). 30840423

[20] Ngoc CT (2018). Conclusions of the digital health hub of the Transform Africa Summit (2018): Strong government leadership and public-private-partnerships are key prerequisites for sustainable scale up of digital health in Africa. BMC Proc..

[21] Robertson C, Sawford K, Daniel SLA, Nelson TA, Stephen C (2010). Mobile phone-based infectious disease surveillance system, Sri Lanka. Emerg. Infect. Dis..

[22] Mtema Z (2016). Mobile phones as surveillance tools: Implementing and evaluating a large-scale intersectoral surveillance system for rabies in Tanzania. PLoS Med..

[23] Abaza H, Marschollek M (2017). mHealth application areas and technology combinations. A comparison of literature from high and low/middle income countries. Methods Inf. Med..

[24] Karimuribo ED (2017). A smartphone App (AfyaData) for innovative One Health disease surveillance from community to national levels in Africa: Intervention in disease surveillance. JMIR Public Health Surveill..

[25] Beyene TJ (2018). A smartphone-based application improves the accuracy, completeness, and timeliness of cattle disease reporting and surveillance in Ethiopia. Front. Vet. Sci..

[26] Mendoza, G., Okoko, L., Konopka, S. & Jonas, E. *mHealth Compendium, Volume Three*. 1–69 (African Strategies for Health project, Management Sciences for Health, 2013).

[27] Singh Y, Jackson D, Bhardwaj S, Titus N, Goga A (2019). National surveillance using mobile systems for health monitoring: Complexity, functionality and feasibility. BMC Infect. Dis..

[28] Mazeri S (2021). Using data-driven approaches to improve delivery of animal health care interventions for public health. Proc. Natl. Acad. Sci. USA.

[29] Food and Agricultural Organization (FAO). *FAO Supports Kenya to Address Zoonotic Diseases and Animal Health*. http://www.fao.org/ag/againfo/home/en/news_archive/2017_FAO_supports_Kenya.html (2017).

[30] Oyas H (2018). Enhanced surveillance for Rift Valley Fever in livestock during El Niño rains and threat of RVF outbreak, Kenya, 2015–2016. PLoS Negl. Trop. Dis..

[31] Thumbi SM (2019). Mobile phone-based surveillance for animal disease in rural communities: Implications for detection of zoonoses spillover. Philos. Trans. R. Soc. Lond. B. Biol. Sci..

[32] Njenga K (2020). High real-time reporting of domestic and wild animal diseases following rollout of mobile phone reporting system in Kenya. Preprint.

[33] Cardona GA, Carmena D (2013). A review of the global prevalence, molecular epidemiology and economics of cystic echinococcosis in production animals. Vet. Parsitol..

[34] Macpherson CN (1985). Epidemiology of hydatid disease in Kenya: A study of the domestic intermediate hosts in Masailand. Trans. R. Soc. Trop. Med. Hyg..

[35] Mungube EO (2006). The prevalence and economic significance of *Fasciola gigantica* and *Stilesia hepatica* in slaughtered animals in the semi-arid coastal Kenya. Trop. Anim. Health Prod..

[36] Kithuka JM, Maingi N, Njeruh FM, Ombui JN (2002). The prevalence and economic importance of bovine fasciolosis in Kenya—An analysis of abattoir data. Onderstepoort J. Vet. Res..

[37] Owiny, M.O., Obonyo, M.O., Gatongi, P.M. & Fèvre, E.M. Prevalence and spatial distribution of *Trematode cercariae* in vector snails within different Agro-Ecological zones in Western Kenya. *Pan. Afr. Med. J.* **32,** 142. 10.11604/pamj.2019.32.142.14418 (2019). 10.11604/pamj.2019.32.142.14418 PMC6607274 31303914

[38] Froyd G (1960). The incidence of liver flukes (*Fasciola gigantica*) and hydatid cysts (*Echinococcus granulosus*) in Kenya cattle. J. Parasitol..

[39] Wahlers K (2012). Cystic echinococcosis in sub-Saharan Africa. Lancet Infect. Dis..

[40] Kia EB, Ouma FF, Mulambalah CS, Okoth PK (2019). The burden of cystic echinococcosis in Kenya: A review article. Iran J. Parasitol..

[41] Mutua F (2018). Piloting a livestock identification and traceability system in the northern Tanzania-Narok-Nairobi trade route. Trop. Anim. Health Prod..

[42] Matete, G.O., Maingi, N., Muchemi, G., Ogara, W. & Gathuma, J.M. Design and development of an electronic identification and traceability system for cattle under pastoral production systems: A case for Kenya. *Livestock Res. Rural Dev.* http://www.lrrd.cipav.org.co/lrrd22/8/mate22139.htm (2010).

[43] Torgerson PR (2017). zDALY: An adjusted indicator to estimate the burden of zoonotic diseases. One Health..

[44] Rushton J (2018). Initiation of global burden of animal diseases programme. Lancet.

[45] Otte, M.J., Chilonda, P. *Cattle and Small Ruminant Production Systems in Sub-Saharan Africa. A Systematic Review*. http://www.fao.org/ag/againfo/resources/en/publications/agapubs/AGAL-Y4176E.pdf (Food and Agriculture Organization of the United Nations, 2002).

[46] Inter-Government Authority on Development [IGAD]. *The Contribution of Livestock to Kenyan Economy. IGAD Livestock Policy Initiative Working Paper No. 03-11*. https://cgspace.cgiar.org/bitstream/handle/10568/24972/IGAD_LPI_WP_03-11.pdf?sequence=1 (2011).

[47] Swai ES, Hayghaimo AA, Hassan AA, Mhina BS (2015). The slaughter of increased numbers of pregnant cows in Tanga abattoir, Tanzania: A cause for concern?. Onderstepoort J. Vet. Res..

[48] Maurer P, Lücker E, Riehn K (2016). Slaughter of pregnant cattle in German abattoirs—Current situation and prevalence: A cross-sectional study. BMC Vet. Res..

[49] European Food Safety Authority [EFSA] Animal Health and Animal Welfare Panel *et al*. Scientific opinion on the animal welfare aspects in respect of the slaughter or killing of pregnant livestock animals (cattle, pigs, sheep, goats, horses). *EFSA J*. **15,** 4782. 10.2903/j.efsa.2017.4782 (2017). 10.2903/j.efsa.2017.4782 PMC7009911 32625488

[50] Okorie-Kanu, O.J. *et al*. Slaughter of pregnant animals for meat at Nsukka slaughterhouse and its economic implications: A public health concern. *Vet. World*. 10.14202/vetworld.2018.1139-1144 (2018). 10.14202/vetworld.2018.1139-1144 PMC6141302 30250375

[51] Nielsen SS, Sandøe P, Kjølsted SU, Agerholm JS (2019). Slaughter of pregnant cattle in Denmark: Prevalence, gestational age, and reasons. Animals.

[52] Calain P (2007). From the field side of the binoculars: A different view on global public health surveillance. Health Policy Plan..

[53] Halliday J (2012). Bringing together emerging and endemic zoonoses surveillance: Shared challenges and a common solution. Philos. Trans. R. Soc. Lond. B Biol. Sci..

[54] Biotechnology and Biological Sciences Research Council. *Case Study: Public Engagement Paves the Path to Impact in Kenya*. https://bbsrc.ukri.org/documents/case-study-public-engagement-paves-the-path-to-impact-in-kenya1/ (2019).

[55] R Core Team. *R: A Language and Environment for Statistical Computing*. https://www.R-project.org/ (R Foundation for Statistical Computing, 2020).

[56] Hyndman RJ, Khandakar Y (2008). Automatic time series forecasting: The forest package for R. J. Stat. Softw..

[57] Kleiber & C., Zeileis, A. *Applied Econometrics with R*. 1–222 (Springer, 2008).

[58] Venables, W.N. & Ripley, B.D. *Modern Applied Statistics with S*. 4th edn. 1–498 (Springer, 2002).

[59] Zeileis A, Hothorn T (2002). Diagnostic checking in regression relationships. R. News.

[60] Hothorn T, Bretz F, Westfall P (2008). Simultaneous inference in general parametric models. Biometric. J..

